# Potential uses of auditory nerve stimulation to modulate immune responses in the inner ear and auditory brainstem

**DOI:** 10.3389/fnint.2023.1294525

**Published:** 2023-12-14

**Authors:** Benjamin J. Seicol, Zixu Guo, Katy Garrity, Ruili Xie

**Affiliations:** ^1^Department of Otolaryngology, The Ohio State University, Columbus, OH, United States; ^2^Department of Neuroscience, The Ohio State University, Columbus, OH, United States

**Keywords:** nerve stimulation, inflammation, bioelectronic medicine, auditory nerve, macrophages, microglia, cochlea, cochlear nucleus

## Abstract

Bioelectronic medicine uses electrical stimulation of the nervous system to improve health outcomes throughout the body primarily by regulating immune responses. This concept, however, has yet to be applied systematically to the auditory system. There is growing interest in how cochlear damage and associated neuroinflammation may contribute to hearing loss. In conjunction with recent findings, we propose here a new perspective, which could be applied alongside advancing technologies, to use auditory nerve (AN) stimulation to modulate immune responses in hearing health disorders and following surgeries for auditory implants. In this article we will: (1) review the mechanisms of inflammation in the auditory system in relation to various forms of hearing loss, (2) explore nerve stimulation to reduce inflammation throughout the body and how similar neural-immune circuits likely exist in the auditory system (3) summarize current methods for stimulating the auditory system, particularly the AN, and (4) propose future directions to use bioelectronic medicine to ameliorate harmful immune responses in the inner ear and auditory brainstem to treat refractory conditions. We will illustrate how current knowledge from bioelectronic medicine can be applied to AN stimulation to resolve inflammation associated with implantation and disease. Further, we suggest the necessary steps to get discoveries in this emerging field from bench to bedside. Our vision is a future for AN stimulation that includes additional protocols as well as advances in devices to target and engage neural-immune circuitry for therapeutic benefits.

## 1 Introduction

Hearing loss is a common condition affecting more than 14% of individuals in the US or over 38 million people (Goman and Lin, 2016). The prevalence increases dramatically with age, where 25% of adults in their sixties and almost 2 in 3 people over the age of seventy develop age-related hearing loss (ARHL) (Goman and Lin, 2016). The number will likely continue to rise with increasing life expectancy and may reach 73 million in the US by 2060 (Goman et al., 2017). Treatment of hearing loss usually starts with hearing aids to increase the intensity of incoming sounds. Despite potential improvements in quality of life, including social engagement and communication (Davis et al., 2016), hearing aid use does not prevent the progression of hearing loss during aging (Dunya et al., 2021), which increases the risk of dementia among elderly with moderate to severe hearing loss (Huang et al., 2023). Given the limitation to prevent hearing loss progression, it is imperative to better understand the mechanisms underlying hearing loss development and explore new potential interventions to improve patient outcomes.

**Figure 1:**
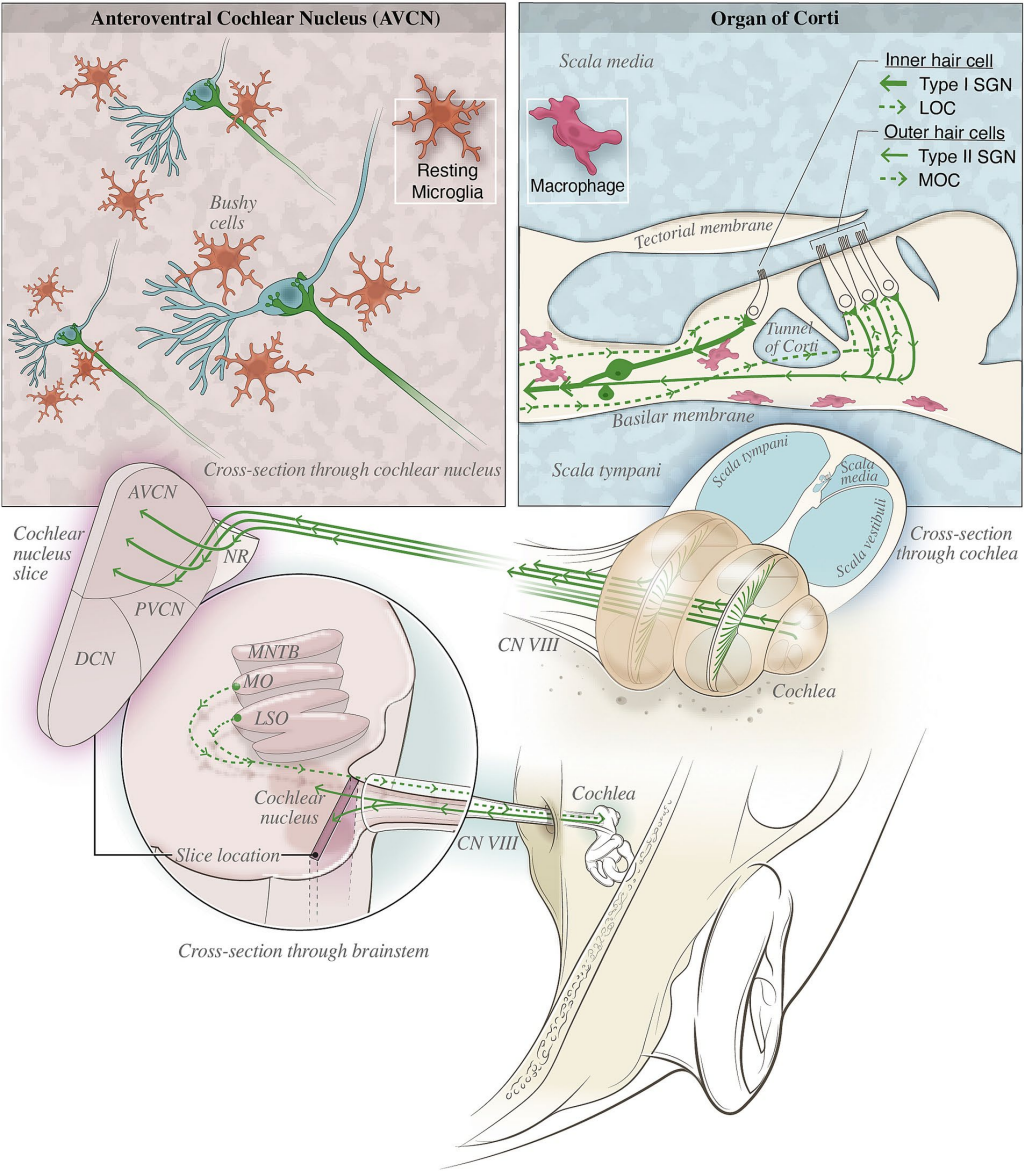
Illustration depicting various macrophage populations and nearby nerves in the organ of Corti and cochlear nucleus (CN). (Top left) depicts the neural-immune interactions likely in the CN where microglia (orange), shown in their resting state, surveil the tissue and promote homeostasis. Once activated, these microglia may aberrantly prune auditory synapses such as the endbulb of Held synapse shown. (Top right) Type II afferent fibers and MOC/LOC efferents may provide neural modulation of inflammation by the release of immunomodulatory neuropeptides such as CGRP. The proximity of these fiber terminals with macrophages (pink) in the basilar membrane could provide the necessary neural-immune communication for bioelectronic medicine treatments to reduce inflammation in the cochlea. (Bottom) shows the anatomical locations of the auditory nerve fibers and their relevant targets within the inner ear and brainstem.

The primary etiology of most hearing loss involves the loss of sensory hair cells and auditory neurons in the cochlea (termed sensorineural hearing loss, or SNHL), including the loss of vulnerable cochlear synapses between hair cells and spiral ganglion neurons (SGN) (Kujawa and Liberman, 2015; Liberman, 2017). Such tissue loss occurs across various time windows, from hours and days after acute insults like noise and ototoxic drugs, to months and years of slow degeneration during aging. In consequence, tissue damages in the cochlea lead to reduced sensory input to the auditory brain, with the direct target being the cochlear nucleus (CN). Subsequent structural and functional changes of the central auditory neural network collectively contribute to the progression of hearing loss. The close association of peripheral and central changes during hearing loss was demonstrated in the CN, where central axons of type I SGNs of the auditory nerve (AN) form giant synapses, called endbulbs of Held, onto bushy cells of the CN (shown in the top left panel of Figure 1). These synapses undergo morphological degeneration and functional decline during various forms of hearing loss (O’Neil et al., 2011; Xie, 2016; Xie and Manis, 2017; Zhuang et al., 2017; Wang et al., 2021). Since endbulb of Held synapses specialize in transmitting temporal information of sound crucial for auditory function, the observed changes in their morphology and physiological properties as well as associated changes in postsynaptic bushy neurons (Wang et al., 2023) substantiate their contribution to the development of hearing loss. Taken together, the effective strategy to prevent hearing loss may lie in approaches to reduce tissue damages in the cochlea and to ameliorate the detrimental changes in the central auditory system.

Recent findings have shown that the pathophysiology of hearing loss involves inflammatory responses (Kalinec et al., 2017; Frye et al., 2018; Hu et al., 2018; He et al., 2020; Wang et al., 2023), particularly on both sides of the AN, including the cochlea (Wood and Zuo, 2017; Noble et al., 2019) and the CN (Seicol et al., 2022). In general, inflammation is activated after tissue damage and is characterized by redness, swelling, heat and pain in affected area (Chen et al., 2017). In most tissues throughout the body, unique innate immune cells reside in the tissue to provide surveillance and host defense. In the cochlea, long-lived macrophages perform these functions, migrate to the tissue during embryonic development, and are capable of self-renewal and innate immune memory (Zhang et al., 2021; Hough et al., 2022). Microglia are the tissue resident macrophages of the brain, including in the CN, and play a critical role in the proper wiring of brain circuits during development (Schafer et al., 2012; Milinkeviciute et al., 2019; Chokr et al., 2022). Reactivation of these developmental functions may contribute to age-related diseases, such as Alzheimer’s disease (Hong et al., 2016). During NIHL, highly-activated macrophages in the cochlear enter the basilar membrane near the sensory hair cells and cochlear synapses (He et al., 2020) and may release factors that worsen the tissue damage. Microglia in the CN serve an analogous role (Lawson et al., 1990; Yin et al., 2017; Lee et al., 2021). During aging, macrophages in the cochlea and microglia in the auditory brainstem show patterns of activation consistent with chronic inflammation in their respective niches (Noble et al., 2019; Seicol et al., 2022). Different macrophage morphologies have been observed during homeostasis, suggesting these cells perform various functions (Lendeckel et al., 2022). Investigations into the mechanisms of the activation of these immune cells, along with infiltrating immune cells, continue to shed light on the importance of immune responses in hearing loss (Fujioka et al., 2014; Wood and Zuo, 2017; Frye et al., 2019). It is worthing noting that in both the cochlea and brain, immune activation can have both beneficial and detrimental impacts (DiSabato et al., 2016; Zhang et al., 2021), so harnessing the protective immune responses and reducing the harmful inflammation could improve outcomes in SNHL. In practice, steroids and other immunosuppressive therapies are used to care for patients who present with idiopathic sudden sensorineural hearing loss (Anyah et al., 2017; Murray et al., 2022) or autoimmune inner ear disease (Ciorba et al., 2018), which demonstrate the contribution of the immune system during SNHL. However, there are significant side effects and clinical concerns about the use of steroids in hearing health disorders, and more targeted therapies are needed to improve clinical outcomes for patients (Chandrasekhar et al., 2019; Murray et al., 2022).

One exciting potential method to reduce inflammation in peripheral and central tissues is to use endogenous neural-immune regulation through electrical stimulation of the nervous system (Eberhardson et al., 2020). This approach is used in the field of Bioelectronic Medicine to reduce inflammation and mitigate disease. In the following sections we will first explore the native auditory circuitry that may modulate inflammation (section 3.1) and then propose potential strategies for developing bioelectronic medical therapies in the treatment of inflammation of the auditory system associated with hearing health disorders (section 3.2). In section 3.3, we will highlight current and future technologies capable of AN stimulation that could be used to achieve these goals.

## 2 Uses of AN stimulation to modulate inflammation

### 2.1 Bioelectronic medicine: stimulating nerves to modulate inflammation

Bioelectronic medicine is a rapidly expanding and transformative approach to stimulate neural circuits to restore organ function throughout the body (Ulloa et al., 2017; Peeples, 2019). It encompasses various nerve stimulating paradigms, including cardiac rhythm management, deep brain stimulation and stimulation of the vagus nerve (Pavlov and Tracey, 2022). For example, vagus nerve stimulation (VNS) was shown to regulate immune functions, and was used to treat rheumatoid arthritis, diabetes, inflammatory bowel disease, endotoxemia, and septic shock through the activation of neural-immune circuits (Ulloa, 2005; Huston et al., 2006; Gautron et al., 2015; Baral et al., 2019; Liu et al., 2021; Tanaka et al., 2021; Pavlov and Tracey, 2022). VNS has also been applied to some hearing health disorders, including tinnitus and vestibular migraine (Chen et al., 2017; De Ridder et al., 2021). The efficacy of VNS in treating tinnitus remains unclear (Stegeman et al., 2021), but was shown to be beneficial for migraine, although the underlying mechanisms need further investigation. Transcutaneous VNS (tVNS) of the auricular branch of the vagus nerve (ABVN) provides a non-invasive method for activating vagal afferents and may be safer and effective in the treatment of migraine and tinnitus (Butt et al., 2020). Better understanding the specific neural circuits activated by VNS could greatly improve the value of this new approach in treating hearing health and vestibular disorders.

One potential mechanism of bioelectronic medicine is that nerve stimulation may indirectly act on the immune system and modulate its activity via the release of acetylcholine and activation of alpha-7 nicotinic acetylcholine receptors (α7nAChR) in target tissues (Borovikova et al., 2000; Wang et al., 2003; Rosas-Ballina et al., 2011). Immune cells that express α7nAChRs are suppressed by acetylcholine (Sitapara et al., 2020; Chen et al., 2022), leading to reduced inflammation (Koopman et al., 2016; Bonaz et al., 2021). Alternatively, the nervous and immune systems may interact through sensory neurons to modulate local immune responses (Baral et al., 2019). Nociceptors are somatosensory neurons that have extensive innervation throughout the body (Julius and Basbaum, 2001). C-fibers, for example, are thin, unmyelinated, and slow-conducting nociceptors that respond to noxious mechanical force, chemicals, and extreme temperatures (Julius and Basbaum, 2001; Woolf and Ma, 2007). These neurons also respond to immune mediators and directly to pathogen products (Chiu et al., 2012, 2016). Activation of nociceptors can drive antidromic release of neuropeptides, such as calcitonin gene-related peptide (CGRP) (Chiu et al., 2016), that regulate local immune responses (Jancso et al., 1967). CGRP is typically anti-inflammatory and can suppress macrophage activation (Nong et al., 1989; Yaraee et al., 2005; Baliu-Pique et al., 2014; Mikami et al., 2014; Baral et al., 2018, 2019).

Neural-immune circuits are likely capable of regulating inflammation in the auditory pathway. In the cochlea, for instance, type II SGNs innervate the three rows of outer hair cells and may act as the “auditory nociceptor” (Flores et al., 2015; Liu et al., 2015). These SGNs have thin unmyelinated nerve fibers like C-fibers that respond to noxious noise and report hair cell damage and may regulate inner ear local immune response (Kiang et al., 1982; Asai et al., 2010; Flores et al., 2015; Liu et al., 2015; Long et al., 2018). The presence of immune mediators such as CGRP were observed in type II SGNs, however, the location of release and functional consequences have not been fully addressed (Wu et al., 2018; Vyas et al., 2019). Although the functional relevance between type II afferent and C-fibers remains speculative, their shared characteristics strongly suggest the existence of a similar immune modulatory mechanism in the inner ear. Furthermore, auditory neural-immune circuits may also involve the lateral and medial olivocochlear (LOC and MOC, respectively) efferent pathways (Kiang et al., 1982; Ryugo, 1992). As shown in Figure 1, the LOC and MOC are part of the auditory efferent system that originate in the olivary nuclei of the auditory brainstem and projects to the organ of Corti to modulate the activity of sensory hair cells and the AN fibers (Guinan, 2018). Stimulation of LOC or MOC efferents can result in the release of diverse neurotransmitters and neuromodulators, such as the anti-inflammatory neurotransmitter acetylcholine and CGRP (Maison et al., 2003; Schrott-Fischer et al., 2007). Additionally, endocannabinoid signaling in the cochlea (Ghosh et al., 2021) might modulate inflammatory responses although the circuitry needs to be better mapped for bioelectronic medicine to tap into this system. While the immunomodulatory effect of LOC and MOC efferent systems and type II afferent pathway still requires further investigation, current evidence strongly support the importance of neural modulation in controlling inflammation throughout the body and suggest that we might be able to leverage similar mechanisms in the auditory system.

### 2.2 Stimulating the AN to improve outcomes in hearing health disorders

Treatments for hearing loss primarily aim to improve sound input from the ear to the brain through amplification or transformed signal transduction. Hearing aids are the first line treatment for patients to improve auditory perception, however they do little to prevent the progression of hearing loss, especially during aging. Severe hearing loss, in which patients do not benefit from hearing aids because the peripheral AN is no longer connected to sensory hair cells may require more invasive prosthetics, such as the cochlear implant (CI). In many cases, preserving cochlear health is critical for potential CI patients in order to maintain residual hearing before CI use is even considered. A major challenge is immune rejection and exacerbation of hearing loss severity from the implantation. Given the existence of potential neural-immune circuitry in the cochlea (reviewed above) and in the brain, we propose a new perspective that stimulating the AN and auditory brainstem could be used to improve outcomes for cochlear or auditory brainstem or midbrain implantation procedures. In the cochlea for example, AN stimulation may activate peptidergic nociceptors (type II SGNs) and efferents (LOCs, MOCs). It is conceivable that release of CGRP into organ of Corti spaces can reduce the activation of proximate macrophages (see Figure 1, top right panel) in respective area and better preserve cochlea health and thus hearing outcome. The idea may be counterintuitive and against current protocols in the clinic that CIs are only activated after a long period of recovery from surgery to allow for healing. However, it is particularly intriguing to test the potential benefits of early CI stimulation during the recovery phase. Proof-of-concept studies should be performed to investigate the optimal timing and stimulation paradigm to engage these neural-immune circuits for better tissue preservation in animal models, using direct stimulation through the implants or optogenetic activation of specific AN fibers. If this kind of stimulation works as predicted, it may be possible to enhance healing during this post-surgery recovery period and improve hearing outcome. The viability of this approach may depend on the remaining auditory circuitry, therefore inclusion criteria for future clinical trials should be carefully considered for AN stimulation in patients with CIs. It is also possible that unintentional recruitment of neural-immune circuits occurs in the cochlea under current clinical practice, which may be amended after better understanding of the physiology and function of these pathways, leading to improvements in chronic outcomes like reduced fibrosis and enhanced perception. We suggest that this is a valuable line of new investigations to augment current strategies, including anti-inflammatory coatings and improvements in surgical techniques (discussed in more detail below).

Beyond the CI patient population, we envision many applications of peripheral and central auditory stimulation for patients with hearing health disorders. As described earlier, hearing loss during normal aging and following acoustic trauma is accompanied by dramatic increases in macrophage/microglia activation and inflammation. Microglia prune synapses in an activity-dependent manner (Schafer et al., 2012), so activating afferent AN fibers to drive central processing could reduce microglial activation during aging and ameliorate ARHL. Stimulated release or inhibition of CGRP could also be beneficial for vestibular conditions, including balance disorders and migraines. Non-invasive stimulation, such as transcranial magnetic or direct current stimulation, could be used to activate LOC and MOC fibers and may be able to reduce inflammation under both ARHL and NIHL. Immune changes associated with various hearing health disorders have begun to be elucidated in animal models and clinical studies [reviewed in (Perin et al., 2021)], however, further investigations into these mechanisms are required to better understand the contribution of immune responses to specific conditions such as tinnitus, hyperacusis, or Meniere’s Disease. Tinnitus is a common condition that may respond to AN or cortical stimulation given its association with NIHL and inflammation in the auditory cortex (Wang et al., 2019). Hyperacusis, which is often a refractory condition that can cause severe disability (Liu et al., 2015), may share a similar etiology with other chronic pain conditions (Williams et al., 2021; Coey and De Jesus, 2023) where the activation of macrophages in the dorsal root ganglia (DRG), for example, contribute to the onset and progression of neuropathic pain (Yu et al., 2020; Guimarães et al., 2023). DRG stimulation is an efficacious therapy (Berger et al., 2021; Chapman et al., 2023) suggesting similar benefits may apply to hyperacusis, especially hyperacusis with pain (noxacusis) (Liu et al., 2015; Williams et al., 2021; Coey and De Jesus, 2023). Clinical application of sound therapy for hyperacusis/noxacusis further supports this possibility (Sheppard et al., 2020; Henry, 2022). Meniere’s Disease is likely a cluster of disorders associated with hearing loss, vertigo, tinnitus, and aural fullness (Basura et al., 2020; Perez-Carpena and Lopez-Escamez, 2020), and may be treated with CI to reduce many of these symptoms (Desiato et al., 2021). Recent application of tVNS of the ABVN showed promising results as an adjunctive therapy for patients with Meniere’s Disease (Wu et al., 2023), possibly due to immunological contributions to disease pathology (Flook et al., 2023). Overall, AN stimulation, stimulation of central auditory centers, and tVNS of the ABVN could be valuable approaches to find new therapies for patient populations with various hearing health disorders.

### 2.3 Overview of technology, interfaces, and challenges with auditory stimulation

CIs are the most successful neural prostheses in treating severe hearing loss. The overall performance, however, has reached a plateau after decades of development. The condition of spiral ganglion neurons, the preservation of synaptic function, and the number and survival of inner and outer hair cells have all been shown to be vital in CI efficacy (Schvartz-Leyzac et al., 2023), and all of which are significantly impacted by acute and chronic inflammatory responses due to surgical trauma during insertion (Seyyedi and Nadol, 2014; Ishai et al., 2017) and foreign body reactions after the surgery (Wilk et al., 2016). CIs made with platinum and silicon trigger foreign body reaction to the device due to incompatibility and rigidity, resulting in inflammation, fibrotic scarring, and reduced wound healing (Lotti et al., 2017; O’Malley et al., 2017), and can cause neo-ossification and eventual break down of the device (Foggia et al., 2019). Acute inflammation immediately after implantation can lead to chronic inflammation that is known to worsen the patient’s long-term health outcomes (Anderson et al., 2008). When the function of the cochlea nerve deteriorates to the point the CI is no longer viable, auditory brainstem implants (ABI) are used. Instances where ABIs are needed include ossification, a lack of cochlear nerve present, structural defects, aplasia, or impairment to the cochlea itself. Individuals with neurofibromatosis are the main group that would have difficulties with CIs because tumors develop on their nerve tissue, damaging the areas required for a traditional CI to provide benefit. The auditory-midbrain implant is another device used instead of a traditional CI by stimulating the inferior colliculus (Lim et al., 2009). All these devices could be used to activate neural-immune circuits along the auditory pathway to help reduce inflammation in respective sites with the potential to improve treatment outcome. The type of stimulation capable of eliciting a desired immunological outcome will need to be determined empirically based on both the device design (Ertas et al., 2022) and the physiology of the target neural-immune circuits (e.g., activation of type II SGNs or MOC/LOC efferents), which need to first be better understood (depicted in Figure 1).

Improvements in biocompatibility, including the use of softer materials, along with robotic surgical techniques, will reduce the occurrence of inflammation and foreign body responses. For example, a recent form of CI implantation consists of using a micro-mechanical tool that reduces surgical trauma due to its high precision and low variability (Banakis Hartl et al., 2019). Further advances including CI with surface coating that slowly release anti-inflammatory drugs such as steroids have also shown promising results in improving CI efficacy (Wulf et al., 2022). Beyond these developments, we predict that our proposed strategy of using electrical stimulation to engage neural-immune circuits is one untapped solution with high potential to reduce acute and chronic inflammation and improve the outcomes of hearing loss treatments in patients.

## 3 Discussion

We have outlined the potential mechanisms and possible applications of using AN stimulation to modulate immune responses in the auditory system and provided a framework for basic and translational studies to test the efficacy of such interventions. In addition to our vision described above, we also anticipate future advances in brain-computer interfaces and other technologies in bioelectronic medicine that could take these ideas further. A practical limitation currently, for example in tVNS (Butt et al., 2020), is the difficulty of stimulating only specific nerves or, in the case of invasive VNS, stimulating select fibers. Targeted nerve stimulation capable of stimulating select nerve fibers (Fitchett et al., 2021) could improve the precision of both VNS and AN stimulation to only activate neural-immune circuits and spare sensory fibers, such as type I SGNs. Current VNS methods can cause unwanted side effects and this is a likely limitation for the application of bioelectronic medicine to AN stimulation as well, which may be resolved with future technologies, such as precision devices or optogenetic approaches (Booth et al., 2021). Additionally, better mapping of the anatomical and physiological properties of the type II SGN and the innervating efferent fibers could also guide the application of bioelectronic medicine in auditory neuroscience. Finally, combined techniques involving stem cell regenerative therapies augmented with bioelectronic medicine approaches could potentially restore lost AN function (Tang et al., 2018; Guo et al., 2021; Sekiya and Holley, 2021; Fang et al., 2023). Our vision for the future relies on continuous improvements in both basic understanding of the system and new tools to manipulate the nerve function to produce the desired immunological outcomes in the cochlea and auditory system.

## Data availability statement

The original contributions presented in the study are included in the article/supplementary material, further inquiries can be directed to the corresponding author.

## Author contributions

BS: Conceptualization, Investigation, Visualization, Writing – original draft, Writing – review & editing. ZG: Conceptualization, Investigation, Writing – original draft, Writing – review & editing. KG: Investigation, Writing – original draft, Writing – review & editing. RX: Conceptualization, Funding acquisition, Supervision, Visualization, Writing – review & editing.
